# Interaction of Estradiol and Endoplasmic Reticulum Stress in the Development of Esophageal Carcinoma

**DOI:** 10.3389/fendo.2020.00410

**Published:** 2020-07-22

**Authors:** Chen Wang, Peng Wang, Jun-Chao Liu, Zhen-Ao Zhao, Rui Guo, Ying Li, Ya-Sen Liu, Shu-Guang Li, Zi-Gang Zhao

**Affiliations:** ^1^Institute of Microcirculation, Hebei North University, Zhangjiakou, China; ^2^First Affiliated Hospital, Hebei North University, Zhangjiakou, China

**Keywords:** esophageal cancer, endoplasmic reticulum stress, gender difference, estradiol, EC109 cell

## Abstract

Gender differences in esophageal cancer patients indicate that estradiol may have antitumor effects on esophageal cancer. The initiation of endoplasmic reticulum stress (ERS) can induce apoptosis in esophageal cancer cells. However, it is still unknown whether estradiol inhibits the development of esophageal cancer by activating ERS pathway. In this study, the gender difference in the development of esophageal cancer was observed by analyzing clinical data and the experimental tumor xenografts in mice. Meanwhile, we investigated the mechanism of ERS in estradiol-mediated inhibition of esophageal cancer using esophageal squamous cell carcinoma cell line EC109. The proportion of male patients with esophageal cancer was significantly higher than female patients. Meanwhile, male patients were prone to have adventitial invasion. The weight of transplanted tumors in female mice was significantly smaller than that in male mice. *In vitro* experiments showed estradiol inhibits the viability and migration of EC109 cells by increasing the expression of ERS-related proteins, whereas ERS inhibitor 4-PBA abolished the effects of estradiol. In conclusion, our data demonstrate that sex difference exists in the occurrence of esophageal cancer. Estradiol can inhibit the viability and migration of esophageal cancer cells through the activation of ERS, providing a novel insight for esophageal cancer development, treatment, and prevention.

## Introduction

Esophageal cancer is one of the eight most common cancers in the world (1), with poor prognosis and low long-term survival (2). Esophageal cancers were mainly classified by the tumor–node–metastasis (TNM) grading standard, and surgery is a conventional treatment for most types of esophageal cancer. There are also auxiliary treatment methods such as radiotherapy and chemotherapy (3). However, surgery requires high physical conditions of the patients, and the recovery is slow after operation (4). Radiotherapy and chemotherapy often cause adverse reactions, which affects the function of various tissues and organs of the patients and reduces the repair ability of esophageal mucosa (5). Therefore, it is urgent to explore new therapy methods.

Decades of research found that there are significant gender differences in esophageal cancer among all races and across the world (6). The incidence of esophageal cancer is three to four times more common among male than female individuals, but the exact mechanism is unclear (7). The identified risk factors for esophageal cancer also cannot fully explain this gender difference and may be related to sex chromosome mechanism. However, the epidemiological studies (8–10) and some preclinical studies indicate that sex hormones might play an important role in esophageal cancer. Sex steroids such as estrogens contribute to the physiological maturation and cell proliferation of estrogen-dependent tissues, such as breast, ovary, and endometrium (11, 12). Canceration of these tissues is associated with abnormal changes in sex steroid levels. However, the functions of steroid hormones in esophageal cancer are often ignored, although there are significant gender differences in esophageal cancer patients. Steroids can affect cell behaviors through non-genomic and genomic actions (11). Thus, studying the functions and mechanisms of steroids and steroid antagonists holds great promise for esophageal cancer treatment and prevention. With the latest research, it has been found that overexpressions of estrogen receptor α and β in esophageal malignant tumors are associated with prognosis (6, 13). *In vitro* studies also demonstrated that estrogens have remarkable inhibitory effect on the occurrence of esophageal cancer (14, 15). Although the antitumor effect of estrogens on esophageal cancer has been reported, its molecular mechanism is still unknown.

Endoplasmic reticulum stress (ERS) is a reaction induced by the disorder of Ca^2+^ balance and overload accumulation of protein in endoplasmic reticulum when cells are injured. ERS-induced apoptosis is the third apoptosis pathway in addition to the death receptor- and mitochondrial-mediated apoptosis pathways. Recent studies indicate that ERS plays a key role in tumor progression. The initiation of ERS signaling can induce apoptosis in esophageal cancer cells (16, 17), which may represent a novel insight for the therapeutic intervention of esophageal cancer. Several studies have demonstrated the role of E2 treatment in enhancing ERS in a few tumors (18–20). E2-treated MCF-7 cells showed increased ERS, inflammatory stress response, and apoptosis (21). ERS is the key biological event that determines the fate of cells after E2 treatment. However, whether estrogens inhibit the occurrence of esophageal cancer by interaction with ERS has not been investigated.

Therefore, in this study, we analyzed the age and gender data of patients with esophageal cancer and used the murine xenograft model in both sexes to confirm the gender difference in esophageal cancer. Furthermore, the inhibitory effects of estradiol and ERS in the viability and migration of esophageal cancer cells were verified using cell experiments.

## Materials and Methods

### Clinical Data

The data of 372 patients with esophageal cancer treated in the First Affiliated Hospital of Hebei North University from June 2012 to March 2020 were collected. The diagnosis was confirmed by pathological section analysis after operation, and the classification of esophageal cancer was determined at the same time. The age, sex, and the relationship between gender difference and lymphatic metastasis or adventitial invasion were analyzed.

### Cell Culture

Human esophageal squamous cell carcinoma cell lines EC109 were generously provided by Life Science Research Center of Hebei North University. The cells were cultured in Roswell Park Memorial Institute (RPMI) 1640 medium (Gibco) supplemented with 10% fetal bovine serum (Gibco), penicillin (100 U/ml), and streptomycin (100 U/ml). All cells were maintained in the presence of 5% CO_2_ at 37°C in a humidified atmosphere.

### Xenograft Model Establishment

EC109 cells in exponential stage were collected and centrifuged at 1,000 rpm for 5 min. After two washes with phosphate-buffered saline (PBS), and the cell concentration was adjusted to 5 × 10^7^ cell/ml with RPMI 1640 medium without fetal bovine serum. EC109 cell tumor xenografts were established by subcutaneously injecting 1 × 10^7^ cells into the right flanks of 4- to 6-week-old mice. The tumor-bearing mice were divided into male and female group; each group included eight mice. All procedures were performed under sodium pentobarbital anesthesia. The animal experiment was approved by the Animal Ethics Committee of Hebei North University. After 4 weeks of rearing, mice were sacrificed by cervical dislocation. Tumor tissues were harvested, photographed, and weighed. The tumor inhibition rate of the female group was calculated with the formula as follows: tumor inhibition rate = (average tumor weight in male group – average tumor weight in female group)/average tumor weight in male group ×100%.

### Analysis of Cell Viability

EC109 cells were assigned into the control group (vehicle), E2 group (10 nM), E2 + ICI group (10 nM E2 and 1 μM ICI 182, 780, an estrogen receptor antagonist), E2 + 4-PBA (ERS inhibitor) group (10 nM E2 and 5 mM 4-PBA), and ERS agonist tunicamycin (TM) group (10 μg/ml). Cell Counting Kit-8 (CCK-8, Applygen Technologies Inc.) was used to measure cell viability according to the manufacturer's protocol. In brief, cells growing at the exponential stage were seeded into 96-well plates at a density of 5,000 cells/well in a final volume of 100 μl and exposed to various treatments for 24 h. Ten microliters of CCK-8 solution was added to each well for a 4-h incubation. Cell viability was calculated by measuring the absorbance at 450 nm. All experiments were repeated three times, and the data are expressed as the mean ± SEM of three wells per treatment.

### Analyses of Cell Migration

A cell culture wound-healing assay was performed to analyze cell migration. Cells growing at the exponential stage were seeded into six-well plates at a density of 1 × 10^5^/ml in a final volume of 2 ml. Cells were grown to confluence, and a linear wound was created in the confluent monolayer using a 200-μl micropipette tip. The cells were then washed with PBS to eliminate detached cells. Pictures were taken under the microscope to record the scratches on each well. In order to reduce the effect of DNA replication and proliferation on the cell migration rate, the serum-free medium was used in the current experimentation according to the previous reports (22, 23). After exposure to various treatments for 24 h, the movement of the wound edge was monitored under a microscope (200 ×). The area between the two sides of the scratch is measured using ImageJ software. Cellular migration rate is calculated by the relative area between the two sides of the scratch. The formula of calculation is as follows: cell migration rate = (scratch area before treatment – scratch area after treatment)/(scratch area before treatment) × 100%.

### Analyses of Immunofluorescence

After counting the cells, cells growing at the exponential stage were seeded into confocal dish at a density of 1 × 10^5^/ml in a final volume of 200 μl and cultured for 16–18 h. After cultured in serum-free RPMI 1640 medium for 24 h, the cells were exposed to various treatments for 24 h. Then, cells were washed with PBS and fixed with 4% paraformaldehyde/PBS (30 min) for confocal microscopic analysis. After permeabilization with Triton X-100, cells were blocked with 5% normal bovine serum albumin for 30 min and incubated with antiglucose regulated protein 78 (GRP78) (1:500, ab21685, Abcam), antiestrogen receptor α (ERα) (1:100, ab32063, Abcam), and anti-ERβ (1:100, ab212351, Abcam), respectively, at 4°C overnight. After rinsing with PBS, the dishes were incubated with corresponding fluorescence secondary antibodies for 90 min. The dishes were mounted after staining with 4′,6-diamidino-2-phenylindole (DAPI) for 5 min and analyzed under an Olympus laser confocal microscope (Olympus, Japan).

### Analyses of Western Blotting

EC109 cells were seeded into six-well plates and were treated with different agents for 24 h when the confluence reached 80%. The cells were washed with PBS and lysed in 100 μl radioimmunoprecipitation assay (RIPA). After brief sonication and centrifugation, the supernatants were collected for protein concentration measurement by bicinchoninic acid (BCA) kit. The proteins in each group were separated using sodium dodecyl sulfate–polyacrylamide gel electrophoresis (SDS-PAGE) and transferred to polyvinylidene fluoride (PVDF) membrane. After blocking with 5% milk, the membranes were incubated with primary antibodies (1:1,000), including anti-GRP78 (ab21685, Abcam), antiactivating transcription factor 6 (ATF6) (ab203119, Abcam), anti-inositol-requiring enzyme 1α (IRE1α) (ab37117, Abcam), antiprotein kinase RNA-like endoplasmic reticulum kinase (PERK) (70R-17036, Fitzgerald), and anti-β-actin (E2317, Cell Signaling Technology). After incubation overnight at 4°C, the membranes were washed and incubated with secondary antibodies (diluted 1: 2000) at room temperature for 1 h. Enhanced chemiluminescence (ECL) was used for signal development. BioRad imaging system was used to capture the chemiluminescence. Analysis was conducted using Quantity One software, and the relative protein levels were expressed as the intensity ratios of target protein to β-actin.

### Statistical Analyses

Statistical analysis was performed using SPSS version 22. All experiments were independently performed at least three times. All values are expressed as the mean ± SEM. Differences among groups were analyzed using one-way analysis of variance (ANOVA) by a least significant difference, and *post hoc* test was performed for testing for all data. The related results of xenograft were tested by two independent samples *t*-test. A difference of *P* < 0.05 was considered to be statistically significant.

## Results

### Gender Differences in Esophageal Cancer

**Figure 1:**
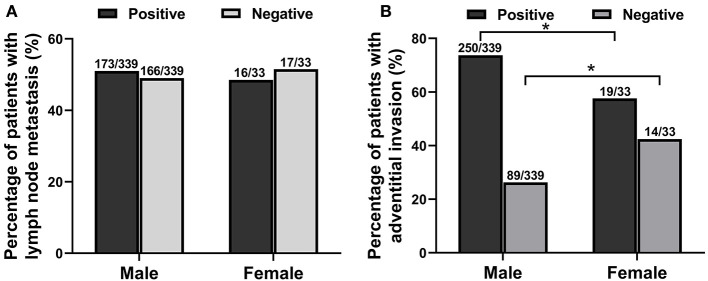
The relationship between gender difference and lymphatic metastasis or adventitial invasion in patients with esophageal cancer from June 2012 to March 2020. **(A)** The relationship between gender and lymphatic metastasis. **(B)** The correlation between gender and adventitial invasion. *P < 0.05, compared with the male group.

Among the 372 esophageal cancer patients, 339 were male (91.13%) and 33 were female (8.87%), with a ratio of male to female of 10.27:1. The proportion of male patients with esophageal cancer was significantly higher than that of female patients (*P* < 0.05). Among the 372 patients, the youngest was 36 years old and the oldest was 80 years old, and the average age of onset in male patients (61.38 ± 8.34) was slightly lower than that in female patients (65.97 ± 7.51, *P* < 0.05). The sex and age information of patients with esophageal cancer is shown in Table 1. The type of esophageal cancer is mainly squamous carcinoma with a percentage of 88.98% (331/372) in total patients, 88.79% (301/339) in male patients, and 90.91% (30/33) in female patients (Table 2). There was no statistic difference in the percentage of squamous carcinoma between the male and female patients (*P* > 0.05) (Table 2). In addition, there was no relationship between gender and lymphatic metastasis (*P* > 0.05) (Figure 1A), but difference was significant in correlation between gender difference and adventitial invasion (*P* < 0.05) (Figure 1B).

**Table 1:** Sex and age distributions in patients with esophageal cancer derived from the First Affiliated Hospital of Hebei North University from June 2012 to March 2020.

**Sex**	**Number of cases (%)**	**Age distribution**	**Average age**
Male	339 (91.13%)	36–80	61.38 ± 8.34
Female	33 (8.87%)	48–79	65.97 ± 7.52
*P*	<0.05		<0.05

*% represents a percentage of the total cases*.

**Table 2:** Type distribution in esophageal cancer patients derived from the First Affiliated Hospital of Hebei North University from June 2012 to March 2020.

**Type of esophageal cancer**	**Male (%)**	**Female (%)**
Squamous carcinoma	301 (88.79%)	30 (90.91%)
Adenocarcinoma	32 (9.44%)	3 (9.09%)
neuroendocrine neoplasm	6 (1.77%)	0 (0.00%)
Total	339	33

*% represents a percentage of the same sex cases*.

### Effect of Gender Differences on Xenograft Tumor in Mice

**Figure 2:**
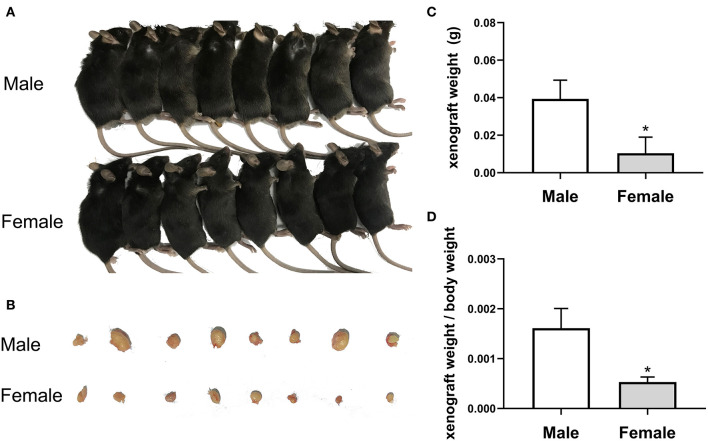
The growth of esophageal cancer xenograft in male and female mice. **(A)** The establishment of xenograft model in mice. **(B)** The appearance of tumor xenograft from the male and female mice. **(C)** The tumor weight analysis. The results are expressed as the means ± SEM, *n* = 8. *P < 0.05, compared with the male group. **(D)** The ratio of tumor weight to body weight. The results are expressed as the means ± SEM, *n* = 8. *P < 0.05, compared with the male group.

The current study investigated the anticancer potential of female patients by establishing a model of EC109 cell xenograft. The results showed that the xenograft weight and the ratio of xenograft weight to body weight in the female mice (0.010375 ± 0.001908 g, 0.00053 ± 0.000101) were significantly lower than that of the male mice (0.039375 ± 0.009952 g, 0.00161 ± 0.000395, *P* < 0.05) (Figures 2A–D). After calculation, the tumor inhibition rate of the female group was as high as 73.65% compared with the male group. These data revealed that tumor growth was lower in female patients (*P* < 0.05), indicating that estradiol may inhibited tumor growth.

### The Expressions of ERs in EC109 Cells

**Figure 3:**
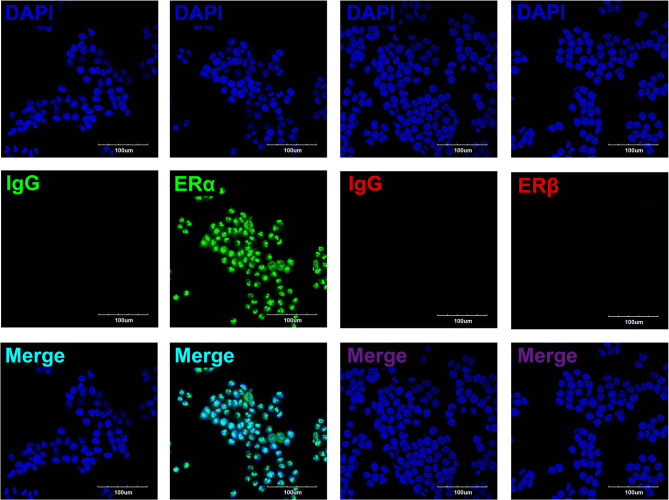
The expressions of estrogen receptors (ERs) in EC109 cells. The representative pictures showed that the positive expression of ERα was located in the nuclei, whereas ERβ was undetectable.

To determine the roles of estradiol in esophageal carcinoma, we detected the protein expressions of ERs in EC109, including ERα and ERβ. Immunofluorescence showed that ERα was expressed in the nuclei of EC109, whereas ERβ was undetectable. These results indicate that estradiol acts mainly through ERα in EC109 (Figure 3).

### Effects of Estradiol on the Migration and Viability of EC109 Cells

**Figure 4:**
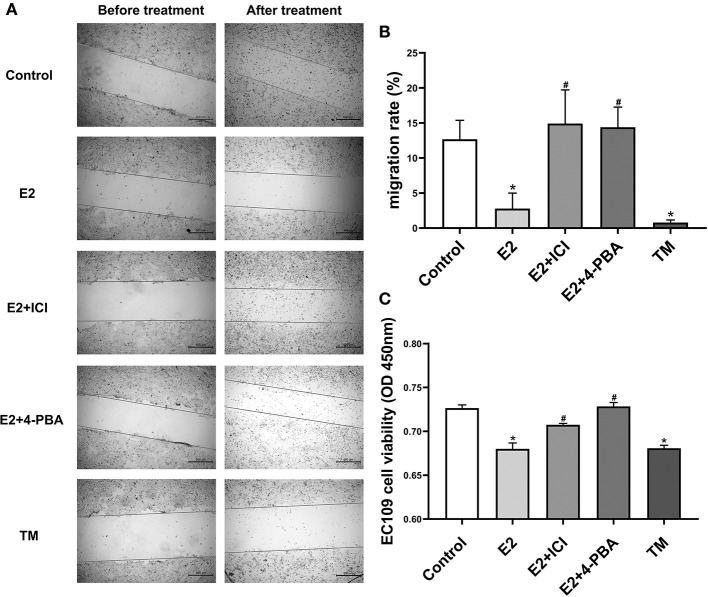
Estradiol treatment reduces the viability and migration rate of esophageal cancer cell line EC109. The cells were cultured in serum-free Roswell Park Memorial Institute (RPMI) 1640 medium for 24 h, and treated with 17-estradiol (E2), E2 and ICI 182,780 (E2 + ICI), E2 and 4-phenylbutyric acid (4-PBA), and tunicamycin (TM) for 24 h, respectively. Then, the cellular migration and proliferation were detected with the cell wound scratch assay and Cell Counting Kit-8 (CCK8) method, respectively. **(A)** Representative wound healing images. The migration rate is calculated by the relative area between the two sides of the scratch. **(B)** The migration rate analysis of EC109 cell. The results are expressed as the means ± SEM, *n* = 3. *P < 0.05, compared with the control group; ^#^*P* < 0.05, compared with the E2 group. **(C)** The EC109 cell viability analysis. The results are expressed as the means ± SEM, *n* = 3. *P < 0.05, compared with the control group; ^#^*P* < 0.05, compared with the E2 group.

To investigate whether estradiol has antitumor effects in esophageal cancer, we attempted to determine whether estradiol affects EC109 cell migration and viability *in vitro*. Estradiol or TM treatment for 24 h significantly decreased cell migration ability (*P* < 0.05), when compared with the control group. On the contrary, estrogen receptor antagonist ICI and ERS inhibitor 4-PBA counteracted the effect of estradiol on the migration ability of EC109 cells (Figures 4A,B). Furthermore, the data showed that both estradiol and TM treatment significantly reduced the viability of EC109 cells compared with the control group, respectively (*P* < 0.05). Meanwhile, ICI and 4-PBA enhanced the viability of EC109 cells after treatment with estradiol (Figure 4C). These results suggested that estradiol may inhibit EC109 cell migration and viability, and this inhibition is mediated by ERS.

### Effect of Estradiol on the Expressions of ERS-Related Proteins in EC109 Cells

**Figure 5:**
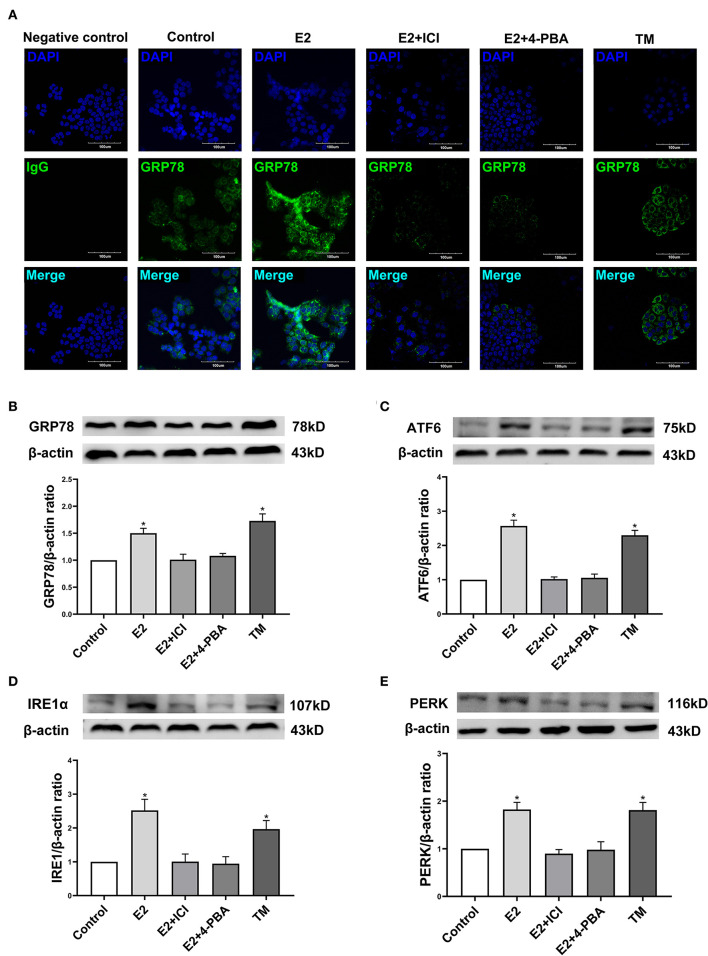
Estradiol treatment upregulates the expression of endoplasmic reticulum stress (ERS) related proteins in esophageal cancer cell line EC109. The cells were cultured in serum-free Roswell Park Memorial Institute (RPMI) 1640 medium for 24 h, and treated with 17-estradiol (E2), E2 and ICI 182,780 (E2 + ICI), E2 and 4-phenylbutyric acid (4-PBA), and tunicamycin (TM) for 24 h, respectively. **(A)** GRP78 proteins in EC109 cells under an Olympus laser confocal microscope. **(B–E)** The expressions of GRP78, ATF6, IRE1α, and PERK in EC109 cells after various treatments. Densitometric values were normalized to β-actin. The results are expressed as the means ± SEM, *n* = 3. *P < 0.05, compared with the control group; ^#^*P* < 0.05, compared with the E2 group. The cells were cultured in serum-free RPMI 1640 medium for 24 h.

In order to further identify the molecular mechanism by which estradiol inhibits the viability of EC109 cells, we analyzed the protein expressions of GRP78, ATF6, IRE1α, and PERK, which were involved in ERS. Immunofluorescence was used to detect the expression of GRP78 in EC109 cells after different treatments. Compared with the control group, GRP78 was greatly increased in cells treated with estradiol and TM. Meanwhile, ICI and 4-PBA treatments significantly decreased GRP78 expression in the E2-treated cells, respectively (Figure 5A). In order to confirm the response of ERS in EC109 cells treated with estradiol, the expressions of ERS-related proteins were detected by Western blotting. Estradiol and TM treatments resulted in the increase in protein expressions of GRP78, ATF6, IRE1α, and PERK in EC109 cells when compared with the control group (*P* < 0.05). Meanwhile, ICI and 4-PBA treatment abolished the estradiol-induced ERS protein expression (*P* < 0.05) (Figures 5B–E). These results suggested that estradiol upregulated ERS in EC109 cells.

## Discussion

There are sex differences in the development of esophageal cancer, and women who underwent resection has a higher overall survival rate than men (9). The known causes (such as smoking, obesity, etc.) cannot well-explain this gender difference. First of all, this study collected and analyzed the clinical data of 372 patients with esophageal cancer treated by surgery in the First Affiliated Hospital of Hebei North University in the past 9 years. The proportion of male patients with esophageal cancer was significantly higher than that of female patients. Next, in this study, we established the xenograft model to verify whether the sex of mice affected the development of esophageal cancer. The results showed that the xenograft weight and xenograft weight/body weight in the female mice were significantly lower than that in the male mice, with a tumor inhibition rate of 73.65% in the female than in the male mice. The results confirm the relation between sex and the development of esophageal cancer.

In light of epidemiological and preclinical studies, more scholars believe that sex hormones may play an important role in the incidence of esophageal cancer (24, 25). Previous report showed that the increased risk of esophageal cancer is related to the decrease in estrogens level (6). Early menopause increases the risk factors for esophageal squamous cell carcinoma (9). It has been shown that premenopausal female patients have a prolonged survival than postmenopausal patients and that female ESCC patients with higher serum estradiol level have a favorable survival rate (10). An epidemiological study of menopausal hormone therapy (MHT) confirmed that ever-users of MHT were at a decreased risk of esophageal adenocarcinoma, gastric adenocarcinoma, and esophageal squamous cell carcinoma. The estrogens-only MHT users had a decreased risk of esophageal and gastric adenocarcinoma in particular (8). The previous study also supported the protective effects of female hormones on the risk of esophageal squamous cell carcinoma (26) and esophageal adenocarcinoma (27). Through several epidemiological studies, the hypothesis of estrogen protection in esophageal cancer has been proposed.

The previous studies found that esophageal squamous cell carcinoma and adenocarcinoma tissues express ERs, including ERα and ERβ (10, 28, 29). Estrogen plays a protective role through ERs, and the patients with esophageal cancer who express ERβ in the nucleus have a better prognosis (10, 28). Some studies have also suggested that ERα plays a protective role in esophageal cancer (12, 30), which is consistent with our data, showing that ERα is highly expressed in EC109 but not ERβ. Esophageal cancer cells also express other sex hormone receptors, such as androgen receptor, progesterone receptor, and so on (30, 31). *In vitro* studies have shown that estrogens have a certain inhibitory effect on cell growth and promote the apoptosis of esophageal cancer cells, which is mediated by the interaction with ERs (32). Only esophageal cancer cells with ERs are inhibited by estrogen, whereas cells without ERs are not (33–35). The Cell Counting Kit-8 (CCK8) results of this study showed that estradiol treatment for 24 h significantly decreased the viability of EC109 cells, and the cell scratch assay demonstrated that the estradiol treatment for 24 h significantly decreased the migration ability of EC109 cells. ICI, ER antagonist, improved the viability and migration ability of EC109 cells treated with estradiol. The results indicate that estradiol can reduce the viability and cell migration ability of esophageal cancer EC109 cells, which is consistent with the previous studies that estradiol has a certain inhibitory effect on the growth of esophageal cancer cells.

Endoplasmic reticulum is an important organelle in cells. Under a variety of physiological or pathological conditions, various stimuli can cause unfolded or misfolded proteins to gather in the endoplasmic reticulum, which is named ERS. ERS is divided into two stages: the early unfolded protein response (UPR) (36) and the late induction of apoptosis. In recent years, studies showed that the initiation of ERS induces apoptosis in esophageal cancer cells (16), and manipulation of ERS signaling has been identified as a therapeutic target for the esophageal cancer. However, whether estrogens interact with ERS to inhibit the occurrence of esophageal cancer has not been reported. GRP78, as immunoglobulin heavy chain binding protein (Bip), belongs to the heat shock proteins family (37), which localized on the ER membrane of all eukaryotic cells. Under ERS, the expression of GRP78 is increased and plays an important role in cell survival and apoptosis by regulating transmembrane ERS sensor (38). Together, ERS is mainly mediated by endoplasmic reticulum molecular chaperone GRP78 protein. In this study, the results of immunofluorescence and Western blotting showed that estradiol treatment for 24 h significantly increased the expression of GRP78 in EC109 cells. Therefore, ERS response was evident in EC109 cells after estradiol treatment. In addition, our results showed that the viability and migration ability of EC109 cells were significantly decreased after treatment with ERS agonist TM. The results suggest that ERS can inhibit the growth of esophageal cancer cells. Moreover, 4-PBA, an ERS inhibitor, could improve the viability and migration ability of E2-treated EC109 cells. In summary, the inhibitory effect of estradiol on the growth of EC109 cells is partly due to the interaction with ERS.

Studies indicate that ATF6, IRE1α, and PERK were the main signaling molecules of ERS. The activation of these signaling molecules were demonstrated by various stress and lead to ERS and UPR (39). These protein levels can directly or indirectly sense to misfolded proteins in the endoplasmic reticulum (40). In order to further identify the molecular mechanism that estradiol inhibits the viability and migration of esophageal cancer cells by regulating ERS, we detected the expression of ERS-related signaling molecules. Our results showed that both estrogen and TM upregulated the expression of ATF6, IRE1α, and PERK proteins in EC109 cells. In contrast, ICI and 4-PBA treatments eliminated the increase in these proteins induced by estradiol. Recent study (41) showed that the activation of UPR starts from dissociation of GRP78 from ATF6, IRE1, and PERK. If the stress is temporary, the activation of ATF6, IRE1, and PERK can enhance the degradation of unfolded and misfolded proteins through proteasomes. However, if the cells suffer from prolonged or severe stress, additional responses are initiated, involving IRE1/Ask1/JNK, caspase-12/caspase-9/caspase-3, ERK/ATF-4/CCAAT/enhancer-binding protein homologous protein (CHOP) pathways, and these pathways can promote apoptosis (42). This is consistent with our findings that estradiol inhibits the viability and migration of EC109 cells by excessive activation of UPR.

**Figure 6:**
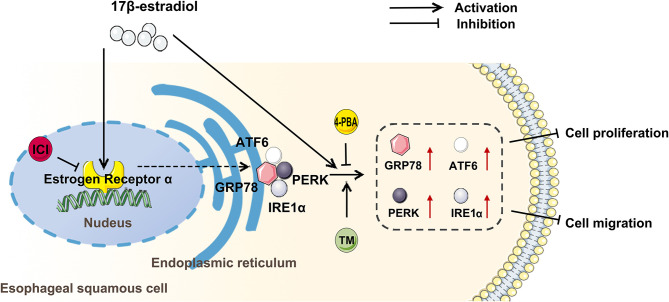
Estradiol treatment inhibits the viability and migration of esophageal cancer cells through the activation of endoplasmic reticulum stress (ERS). ICI, an inhibitor of estrogen nuclear receptors; 4-PBA, an inhibitor of ERS; tunicamycin (TM), an agonist of ERS; GRP78, ATF6, PERK, and IRE1 were ERS-related proteins.

In summary, the present study indicates that the gender difference is involved in the development of esophageal cancer, and estradiol treatment increases the expression of GRP78, ATF6, IRE1, and PERK through estrogen receptor, and upregulates ERS to inhibit the viability and migration of esophageal cancer cells (Figure 6). However, the specific molecular mechanism by which EC109 cell apoptosis is induced by estradiol through upregulating ERS-related pathways still needs to be further studied. It should be pointed out that there were several limitations in the xenograft experiments. The roles of androgens or estradiol, ICI or bicalutamide, and 4-PBA or TM treatments in affecting tumor growth were not investigated, and the sera levels of steroid hormones were not detected in these mice. Therefore, the various treatments and castrated mice should be used to verify the roles of androgens and estradiol in xenograft model in the future study. In addition, an analysis of steroid hormones present in the serum of esophageal cancer patients or mice should be performed in the future. Clarifying the role of steroid hormones in the development of esophageal cancer will make the detection of serum steroid hormone level to be a simple and important tool in the screening and personalized therapy of esophageal cancer.

## Conclusion

There are sex differences in the occurrence of esophageal cancer. Estradiol inhibits the proliferation and migration of esophageal cancer cells by interaction with ERS.

## Data Availability Statement

The datasets generated for this study are available on request to the corresponding author.

## Ethics Statement

The studies involving human participants were reviewed and approved by Medical Ethics Committee of Reproductive Hebei North University. In this retrospective study, we collected and analyzed the age distribution and average age from the 372 patients with esophageal cancer in the first affiliated Hospital of Hebei North University. However, surgery and treatment options for these patients were not involved in current study, and there was no harm or risk to these patients. Therefore, written informed consent for participation was not required for this study in accordance with the national legislation and the institutional requirements.

## Author Contributions

CW, PW, Z-AZ, RG, and YL performed the majority of the animal experiment and laboratory work. CW acquired and analyzed the data. J-CL and Y-SL collected the clinical data. Z-GZ and S-GL involved in the conception and design of the study, data interpretation, and critically revised the manuscript. All authors revised the manuscript critically, approved the version to be published and agreed to be accountable for all aspects of the work.

## Conflict of Interest

The authors declare that the research was conducted in the absence of any commercial or financial relationships that could be construed as a potential conflict of interest.
